# Dr. Frank Harcourt Koyle

**Published:** 1911-03

**Authors:** 


Dr. Frank Harcourt Koyle, of Hornell, N. Y., died at his home January 18, 1911, from pneumonia, aged 46 years. He graduated from the Medical Faculty of Queen's University, Kingston, Ont., in 1888. He was a member of several medical societies, was oculist and aurist to the Steuben Sanitarium, Hornell, and to the Erie Railway Company. His brother, Lieutenant Frederick T. Koyle, is a surgeon in the United States army.

